# Transgenic Overexpression of Galectin-3 in Pancreatic β Cells Attenuates Hyperglycemia in Mice: Synergistic Antidiabetic Effect With Exogenous IL-33

**DOI:** 10.3389/fphar.2021.714683

**Published:** 2021-11-05

**Authors:** Nemanja Jovicic, Ivica Petrovic, Nada Pejnovic, Biljana Ljujic, Marina Miletic Kovacevic, Sladjana Pavlovic, Ilija Jeftic, Aleksandar Djukic, Ivan Srejovic, Vladimir Jakovljevic, Miodrag L Lukic

**Affiliations:** ^1^ Department of Histology and Embryology, Faculty of Medical Sciences, University of Kragujevac, Kragujevac, Serbia; ^2^ Department of Pathophysiology, Faculty of Medical Sciences, University of Kragujevac, Kragujevac, Serbia; ^3^ Department of Immunology, Institute for Biological Research “Siniša Stanković,” University of Belgrade, Belgrade, Serbia; ^4^ Department of Genetics, Faculty of Medical Sciences, University of Kragujevac, Kragujevac, Serbia; ^5^ Center for Molecular Medicine and Stem Cell Research, Faculty of Medical Sciences, University of Kragujevac, Kragujevac, Serbia; ^6^ Department of Physiology, Faculty of Medical Sciences, University of Kragujevac, Kragujevac, Serbia; ^7^ Department of Human Pathology, 1st Moscow State Medical University IM Sechenov, Moscow, Russia

**Keywords:** galectin-3, type 1 diabetes mellitus (T1D), β cells, interleukin 33 (IL-33), regulatory T cells (T reg)

## Abstract

Galectin-3 (Gal-3) has diverse roles in inflammatory and autoimmune diseases. There is evidence that Gal-3 plays a role in both type 1 and type 2 diabetes. While the role of Gal-3 expression in immune cells invading the pancreatic islets in the experimental model of type 1 diabetes mellitus has been already studied, the importance of the overexpression of Gal-3 in the target β cells is not defined. Therefore, we used multiple low doses of streptozotocin (MLD–STZ)–induced diabetes in C57Bl/6 mice to analyze the effect of transgenic (TG) overexpression of Gal-3 in β cells. Our results demonstrated that the overexpression of Gal-3 protected β cells from apoptosis and attenuated MLD–STZ–induced hyperglycemia, glycosuria, and ketonuria. The cellular analysis of pancreata and draining lymph nodes showed that Gal-3 overexpression significantly decreased the number of pro-inflammatory cells without affecting the presence of T-regulatory cells. As the application of exogenous interleukin 33 (IL-33) given from the beginning of MLD–STZ diabetes induction attenuates the development of disease, by increasing the presence of regulatory FoxP3^+^ ST2^+^ cells, we evaluated the potential synergistic effect of the exogenous IL-33 and TG overexpression of Gal-3 in β cells at the later stage of diabetogenesis. The addition of IL-33 potentiated the survival of β cells and attenuated diabetes even when administered later, after the onset of hyperglycemia (12–18 days), suggesting that protection from apoptosis and immunoregulation by IL-33 may attenuate type 1 diabetes.

## Introduction

Type 1 diabetes mellitus (T1DM) is a chronic autoimmune disease characterized by insulin deficiency and increased blood glucose levels as a consequence of the loss of pancreatic islet β cells (reviewed in Katsarou et al., 2017). The pathogenesis of the disease is heterogeneous, and multiple genetic and environmental factors are reported to play a role (Ilonen et al., 2019). The pathogenesis is thought to involve T cell–mediated destruction of β cells, predominantly by autoreactive CD8^+^ T cells (Roep, 2008; Tsai et al., 2008). Tolerogenic dendritic cells and T-regulatory cells are found to downregulate type 1 diabetes (Phillips et al., 2019). The number of regulatory FOXP3^+^ cells in the islets, during insulitis, is very small (Willcox et al., 2009; Gaglia et al., 2011). The main mechanism of β-cell death observed in T1DM rodent models is apoptosis (Eizirik and Mandrup–Poulsen, 2001). Although not completely understood, it appears to involve the interaction of CD8^+^ T cells and β cells *via* Fas ligand and its receptor, and an influx of macrophages and pro-inflammatory CD4^+^ T lymphocytes (Cnop et al., 2005). Secretion of pro-inflammatory cytokines, including interleukin-1β (IL-1β), interferon-γ (IFN-γ), and tumor necrosis factor-α (TNF-a) by the diverse immune cells infiltrating the islet, is accompanied with the production of reactive oxygen species such as nitric oxide (NO) by the macrophages, dendritic cells, and the β cells themselves contributing to the destruction of β cells (Lukic et al., 1991; Cnop et al., 2005).

Galectin-3 (Gal-3) belongs to an evolutionary conserved family of b-galactoside–binding lectins with multifunctional properties expressed in a variety of tissues and cell types. It is primarily localized in epithelial and endothelial cells, as well as in the immune cells, and play a role in multiple inflammatory diseases (reviewed in Srejovic and Lukic, 2021). It is present mainly in the cytoplasm but may also be found in the nucleus, on the cell surface, and in the extracellular environment (Almkvist and Karlsson, 2002). It contains one carbohydrate-binding site (CRD) region coupled to a non-lectin–amino-terminal chain of about 120 amino acid lengths with short repeating sequences rich in proline and glycine. Gal-3 is the only galectin that can merge into pentamers, and in this way, cross-link the surface glycoconjugates by forming a lattice structure (Almkvist and Karlsson, 2002; Ochieng et al., 2002; Liu and Rabinovich, 2005; Pearl-Yafe et al., 2007). Intracellular Gal-3 binds to the ligand *via* the C-terminal domain to protect the cells from apoptosis (Akahani et al., 1997).

Gal-3 appears to be a pro-inflammatory molecule in several inflammatory and autoimmune diseases (de Oliveira et al., 2015). In the T1DM expression of Gal-3 on accessory cells in particular dendritic cells, it appears to lead to the activation of inflammatory lymphocytes and macrophages and causing islet inflammation (Mensah-Brown et al., 2009). It has also been shown that circulatory Gal-3 has a pro-inflammatory effect (Li et al., 2016).

In contrast, intracellular Gal-3 has an anti-apoptotic effect, blocking the pro-apoptotic Bax molecule through the interaction with an anti-apoptotic Bcl-2 in human thyroid carcinoma cells (Harazono et al., 2014). Ikemori et al. (2014) have shown that the enhanced expression of Gal-3 represents an adaptive cell response to stress that prolongs their survival.

While the role of Gal-3 expression in invading immune cells in the experimental model of T1DM has been studied already, the importance of overexpression of this molecule in the target β cells is not defined. Therefore, we used a transgenically enhanced Gal-3 expression in β cells to analyze the effect of intracellular Gal-3 in MLD–STZ–induced diabetes in C57Bl/6 mice. Our results demonstrated that the overexpression of Gal-3 by protecting β cells from apoptosis attenuates MLD–STZ–induced hyperglycemia, glycosuria, and ketonuria. The cellular analysis of the pancreata and draining lymph nodes showed a significant decrease in the number of pro-inflammatory cells without affecting the presence of T-regulatory cells.

Interleukin 33 (IL-33) is a member of the IL-1 family of cytokines expressed constitutively on endothelial and epithelial cells. During inflammation, released upon tissue damage, it stimulates IL-33 receptor (ST2 receptor) expression cells. The main target of IL-33 are mast cells, group 2 innate lymphoid cells, and regulatory T cells (reviewed in Cayrol and Girard, 2018).

We have shown recently that the application of exogenous IL-33 given from the beginning of MLD–STZ diabetes induction attenuates the development of diabetes, by increasing the presence of regulatory FoxP3^+^ ST2^+^ cells (Pavlovic et al., 2018). Therefore, we evaluated the potential synergistic effect of the exogenous IL-33 and TG overexpressed Gal-3 in β cells on disease development. The addition of IL-33 in TG mice further attenuated diabetes even when administered later, after the onset of hyperglycemia, 12–18 days after disease induction.

## Materials and Methods

### Experimental Mice

**FIGURE 1:**
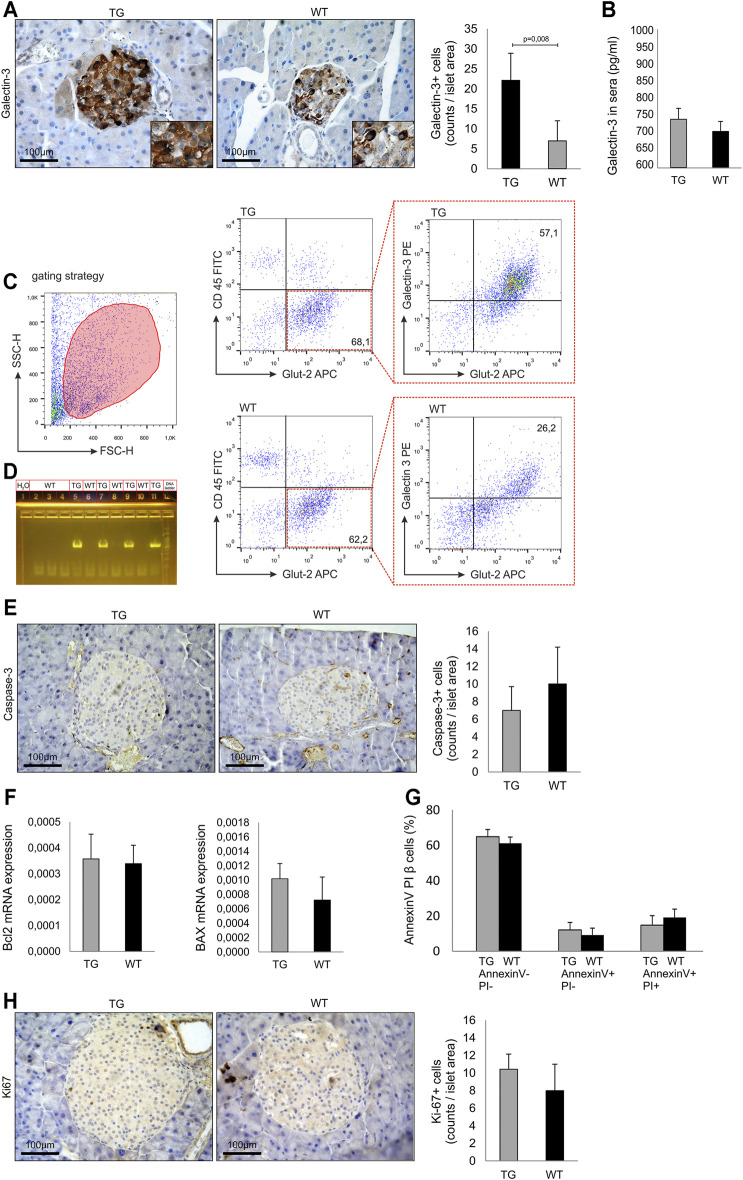
Baseline analysis and islets biology in untreated mice. **(A)** Immunohistochemical expression of galectin-3+ cells in islets of untreated TG and WT mice. **(B)** Level of secreted galectin-3 in sera. **(C)** Representative dot plots of intracellular galectin-3 expression in Glut-2+ beta cells. **(D)** PCR confirmation of genotype. PCR reaction was performed using specific set of primers (Kapa Biosystems, USA), and the presence of a product was visualized on agarose gel. **(E)** Immunohistochemical expression of caspase-3+ cells in the islets of untreated TG and WT mice. **(F)** RT-PCR analysis of relative expression of Bcl-2 and Bax mRNA. **(G)** Annexin V/propidium iodide analysis of isolated islets. **(H)** Immunohistochemical expression of KI-67+ cells in islets of untreated TG and WT mice. The analysis was performed by a light microscope using a magnifying lens of 40 X. Data from two experiments with 5–7 mice per group are shown as mean ± SEM; by the Mann–Whitney U test and independent-sample Student’s *t*-test.

We have used 10–12 weeks old C57Bl/6 male mice (WT) and C57Bl/6 mice with transgenically enhanced Gal-3 expression in pancreatic β cells. Mice with overexpression of Gal-3 in pancreatic β cells were generated and kindly donated by Prof. Bernard Thorens (Center for Integrative Genomics, University of Lausanne, Switzerland) (Thorens et al., 2000). As previously reported (Petrovic et al., 2020), for testing the mice genotype, we extracted DNA from the ear tissue (KAPA Express Extract, KK7102, Kapabiosystems, USA). For the PCR reaction, we used a KAPA 2G Fast Ready Mix PCR Kit (KK5102, Kapabiosystems, USA) and primers listed in Supplementary Material S1. Overexpression of Gal-3 in the pancreatic β cells was confirmed with a 591-bp PCR product visualized on agarose gel (Figure 1D). All mice in both groups received STZ for five consecutive days at a dose of 40 mg/kg ip. The animals were kept in our animal facilities under standard laboratory conditions in a controlled environment with a 12-h light/darkness cycle and received water and standard diet *ad libitum*. The mice were euthanized by cervical dislocation, and blood and the pancreata were sampled for further analyses.

### IL-33 Application

The mice received exogenous mouse IL-33 (0.4 μg/injection; eBioscience) intraperitoneally on the 12th, 14th, 16th, and 18th day after the disease induction. The control animals were treated intraperitoneally with PBS + citrate buffer (CB) solution or IL-33 + citrate buffer solution at the same time interval.

### Ethics Statement

The study was conducted in compliance with all the regulations and recommendations stated in the European Union Directive 2010/63/EU. All animal procedures were approved by the Ethical Committee of the Faculty of Medical Sciences, University of Kragujevac (Permit Number: 01–6675).

### Metabolic Parameters

Body weight and fasting glycemia, ketonuria, and glycosuria were monitored for 21 days, every 72 h. The mice were made to fast for 4 h, and glucose levels (mmol/L) were measured using Accu-Chek Performa Glucometer (Roche Diagnostics, Mannheim, Germany). Ketonuria and glycosuria were measured using qualitative test strips (Roche Diagnostics, Mannheim, Germany). Insulin levels in the serum was measured using the Mouse Insulin ELISA Kit (Cusabio Biotech, Wuhan, China) after euthanizing the animals.

### Histopathological Analyses

Pancreata were excised and placed in 10% buffered formaldehyde fixative solution for 24 h at room temperature. Paraffin-embedded pancreata sections (5 μm) were stained with hematoxylin-eosin and were used for the analysis of mononuclear infiltrates in the pancreatic islets by light microscope (BX51; Olympus, Japan) using a magnification lens of 40 X. The histological analysis of the distribution of inflammatory cell infiltrate in pancreatic islets was performed in blinded fashion by two observers (IP and NJ). Insulitis was graded and a mean insulitis score was calculated as described previously by Hall et al. (2008) according to the following scale: grade 0 (no insulitis) = 0% infiltration within the islets; grade 1 (peri-insulitis) = 1–10% infiltration; grade 2 (moderate insulitis) = 11–50% infiltration; or grade 4 (complete insulitis) = extensive infiltration with few or no detectable pancreatic islet cells. Quantification was performed using ImageJ software ( the National Institutes of Health, Bethesda, MD, USA), on 10 fields/section. Results are presented as a percentage of the affected area of the islet.

### Immunohistochemistry

For immunohistochemical staining, we used paraffin-embedded sections (5 μm) of the mouse pancreas tissue. Deparaffinized tissue sections were incubated with primary mouse anti-Galectin 3 antibody (ab53082, Abcam), anti-Caspase/3 antibody (ab184787, Abcam), anti-Ki-67 (ab16667, Abcam), or insulin antibody (ab63820, Abcam). Staining was visualized by using the mouse-specific HRP/DAB detection IHC kit (ab64259, Abcam), and the sections were counterstained with Mayer’s hematoxylin. The tissue slices were not consecutive, and the analyzed islets were randomly chosen for each parameter. In each analysis, islets with different insulitis score were randomly included. The sections were photomicrographed with a digital camera mounted on a light microscope (Olympus BX51, Japan), digitized, and analyzed. Quantification was performed using ImageJ software (National Institutes of Health, Bethesda, MD, USA), on 10 fields/section. Scoring and histological analysis were performed in blinded fashion by two independent observers. Results are presented as a mean count of positive cells per islet area or the percentage of islet area.

### Isolation of Primary Mouse Islets of Langerhans

Pancreatic islets were isolated from the TG and WT mice by collagenase V digestion technique followed by handpicking (Li et al., 2009). The number of islets isolated by handpicking was in the range from 250 to 300 per animal, and there was no difference between TG and WT mice. Islets were cultured overnight in the RPMI-1640 medium containing 10% fetal calf serum (FCS, PAA Chemicals, Pasching, Austria), 10 mM HEPES, 5 mM 2-mercaptoethanol, 2 mM Lglutamine, 1 mM sodium pyruvate, 100 IU/ml penicillin, and 100 mg/ml streptomycin (culture medium) in a humidified (5% CO, 95% air) atmosphere at 37°C. After recovery, the islets were incubated in the culture medium in 24-well non-adhesive culture plates.

### Flow Cytometry Analysis

Pancreatic draining lymph nodes were removed, and single-cell suspensions were obtained by mechanical disruption. After removing pancreatic lymph nodes, the pancreas was processed through three steps: *in situ* perfusion with collagenase, pancreatic digestion, and isolation of the islets. The cells were separated as described elsewhere and analyzed by flow cytofluorimetry (Li et al., 2009) The isolated cells were stained with either combinations of fluorochrome-labeled primary Abs or isotype controls for 30 min at 4°C. For intracellular staining, the cells were activated with PMA/ionomycin for 4 h and processed as previously described (Foster et al., 2007). For annexin V and PJ staining, the annexin kit was used (4830-01-K, Trevigen, Gaithersburg, Md).

Cells were labeled with fluorochrome-conjugated monoclonal antibodies: anti-mouse CD3 (FAB4841F, R&D Systems, Minneapolis, MN), CD4 (130-102-597, MACS Biotech and FAB554F, R&D Systems, Minneapolis, MN), CD8 (130-102-468, MACS Biotech, Bergisch Gladbach,, Germany and FAB116A, R&D Systems, Minneapolis, MN), CCR6 (FAB5906, R&D Systems, Minneapolis, MN), CXCR3 (562152, BD Biosciences, Franklin Lakes, NJ), FoxP3 (130-093-013, MACS Biotech, Bergisch Gladbach,, Germany), IL-10 (NBP1-00673PCP, NOVUS, Minneapolis, MN), F4/80 (FAB5580A, R&D Systems, Minneapolis, MN), MHCII (12-53322-81, EBioscience, San Diego, CA), IL-17A (130-102-344, MACS Biotech, Bergisch Gladbach,, Germany), INFγ (RM9001, Invitrogen, Waltham, Mass), CD206 (FAB2535, R&D Systems, Minneapolis, MN), TNFα (IC410F, R&D Systems, Minneapolis, MN), IL-1β (IC4013P, R&D Systems, Minneapolis, MN), CD11b (553312, BD Biosciences, Franklin Lakes, NJ), Galectin 3 (130-101-312, MACS Biotech, Bergisch Gladbach,, Germany), CD45 (561088, BD Biosciences, Franklin Lakes, NJ), and GLUT-2 (FAB1440A, R & D Systems, Minneapolis, MN). The cells were analyzed using a FACS Calibur (BD Biosciences, Franklin Lakes, NJ) and FlowJo software (Tree Star, Ashland, OR).

### Statistical Analysis

Statistical analysis was performed using SPSS 22.0. All results are expressed as mean ± SD. Statistical significance was determined by the Mann–Whitney U test, and, where appropriate, student’s independent *t-*test. A *p* value of ≤0.05 was considered statistically significant.

## Results

### TG Overexpression of Gal-3 in β cells Did Not Affect Islets Biology in Untreated Animals

We analyzed the influence of Gal-3 overexpression on islets size, number, and morphology. The approximate number of isolated islets was 250–300 per animal, and there was no difference between TG and WT groups. Also, there was no difference in the average radius of the islets between two cohorts (248 ± 84 μm vs. 257 ± 97 μm, respectively). The isolated islets from both groups had a morphology of healthy islets. Immunohistochemical analysis demonstrated that the number of Gal-3 immunoreactive cells was significantly higher in untreated TG mice than in WT (*p* = 0.008, Figure 1 panel A). There was no difference in the level of secreted Gal-3 in sera (Figure 1 panel B). Flow cytometry analysis demonstrated that the intracellular expression of Gal-3 was significantly higher in Glut-2+ beta cells of TG mice (Figure 1 panel C). Overexpression of galectin 3 in the pancreatic beta cells was confirmed with a 591-bp PCR product visualized on agarose gel (Figure 1 panel D). Next, we analyzed the expression of molecules responsible for apoptosis in untreated animals. There was no difference in the number of caspase-3 immunoreactive cells in the islets (Figure 1 panel E). Likewise, there was no difference in the relative expression of Bcl-2 and BAX mRNA in isolated islets of TG and WT mice (Figure 1 panel F). By the Annexin V/PI staining of cells from isolated islets, we demonstrated that there was no difference between the percentages of viable and apoptotic cells (Figure 1 panel G). Also, there was no difference in the number of Ki-67 immunoreactive cells in the pancreata (Figure 1 panel H).

### TG Overexpression of Gal-3 in β Cells Improves Metabolic Parameters in MLD–STZ–Induced Diabetes.

**FIGURE 2:**
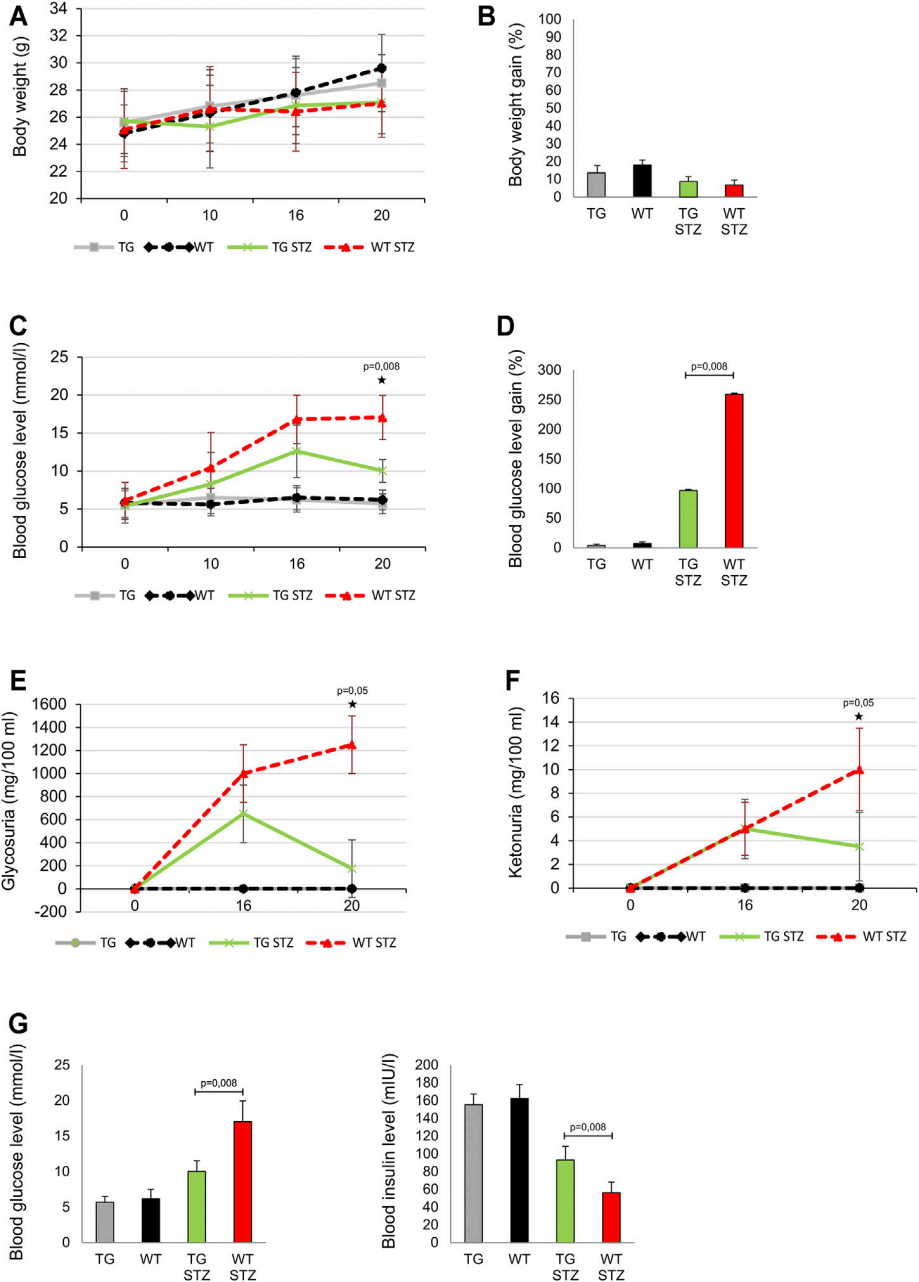
Transgenically enhanced Gal-3 expression on β-cells improves the parameters of glucoregulation in the MLD–STZ model of type 1 diabetes. Differences in the parameters of glucoregulation were observed after the administration of multiple low doses of streptozotocin. **(A)** There were no differences in body weight and **(B)** the body weight gain expressed as percentage of initial body weight. **(C)** On the 20th day of the experiment, glycemia was significantly lower in mice with transgenically enhanced galectin 3 expression on β-cells than in the control group. **(D)** Blood glucose level gain expressed as percentage of initial glycemia was significantly lower in mice with the transgenically enhanced galectin 3 expression on β-cells than in the control group. **(E)** On the 20th day of the experiment, glycosuria and **(F)** ketonuria were significantly lower in TG mice than in the WT mice. **(G)** The blood insulin level was significantly higher in TG mice, while the average glycemia was significantly lower than in the WT group. Data from two experiments with 5–7 mice per group are shown as mean ± SEM; by the Mann-Whitney U test and independent-sample Student’s *t*-test.

We compared MLD–STZ–induced diabetes in WT and the mice with TG overexpressed Gal-3 in β cells. There was no significant difference in body weight and body weight gain between the control TG and WT animals. Similarly, there was no difference in these parameters between two cohorts treated with MLD–STZ (Figure 2 panels A, B). Importantly, on the day 20, at the end of the experiment, blood glucose level was significantly lower in MLD–STZ–treated TG mice than in the treated WT control (Figure 2 panels C, D). By that time, glycosuria and ketonuria were also significantly lower in MLD–STZ–treated TG mice than in the WT mice (*p* = 0.05, Figure 2 panels E, F). As seen in Figure 2, panel G, blood insulin level is inversely correlated with the blood glucose level that was significantly higher in TG mice (*p* = 0.008, Figure 1, panel G).

### TG Overexpression of Gal-3 in β-cells Attenuates Insulitis

**FIGURE 3:**
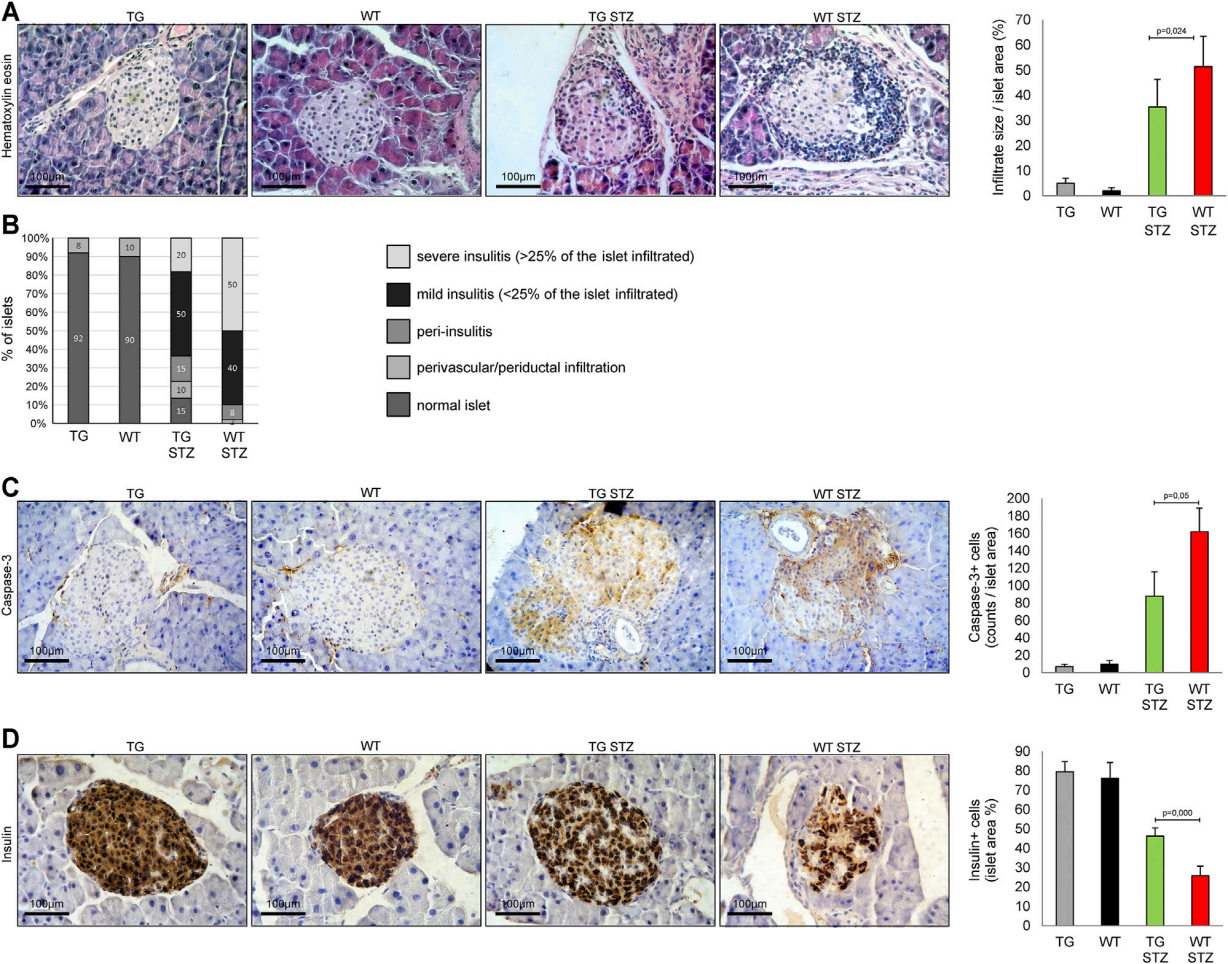
Transgenically enhanced Gal-3 expression in β-cells attenuates inflammation and insulitis. Differences in islet inflammation and insulitis severity were observed after the administration of multiple low doses of streptozotocin. **(A)** Representative photomicrographs of H&E staining. Average size of the mononuclear infiltrate expressed as a percentage of islet area. **(B)** Histology of the islet showed a significantly lower influx of mononuclear cells in TG mice in comparison with the WT group. **(C)** Immunohistochemical expression of caspase-3+ cells in islets was significantly lower in TG STZ mice. **(D)** Immunohistochemical expression of insulin + cells in islets were significantly higher in TG STZ mice. The analysis was performed by a light microscope using a magnifying lens of 40 X. Data from two individual experiments with at least 5–7 mice per group are shown as mean ± SD and compared by the paired *t*-test or Mann–Whitney test.

Figure 1A illustrates the histology of the islets in four groups of animals. The first and second panels of Figure 3A illustrate the structure of normal islets. There was a striking difference between TG and WT animals treated with MLD–STZ. While in TG animals we have seen only single-cell peripheral insulitis, in WT mice, there was a typical accumulation of mononuclear cells as well as single mononuclear cells throughout the islets. Panel B presents the results of the analysis of the number of islets with infiltration of mononuclear cells. This analysis revealed a lower percentage of islets with large infiltrates in TG mice than in WT mice. Therefore, the cumulative average infiltration of mononuclear cells was lower in TG mice than in WT mice (*p* = 0.024, Figure 3 panels A and B). Expression of caspase-3 increased in both groups compared to the untreated control but was significantly lower in TG mice (*p* = 0.05, Figure 3 panel C). Also, the percentage of the islets area immunoreactive to insulin decreased in both treated groups compared to the control and was significantly lower in WT mice than in TG mice (*p* = 0.000, Figure 3 panel D).

### TG Overexpression of Gal-3 in β cells Decreases the Frequencies of Pro-Inflammatory Cells in the Islets

**FIGURE 4:**
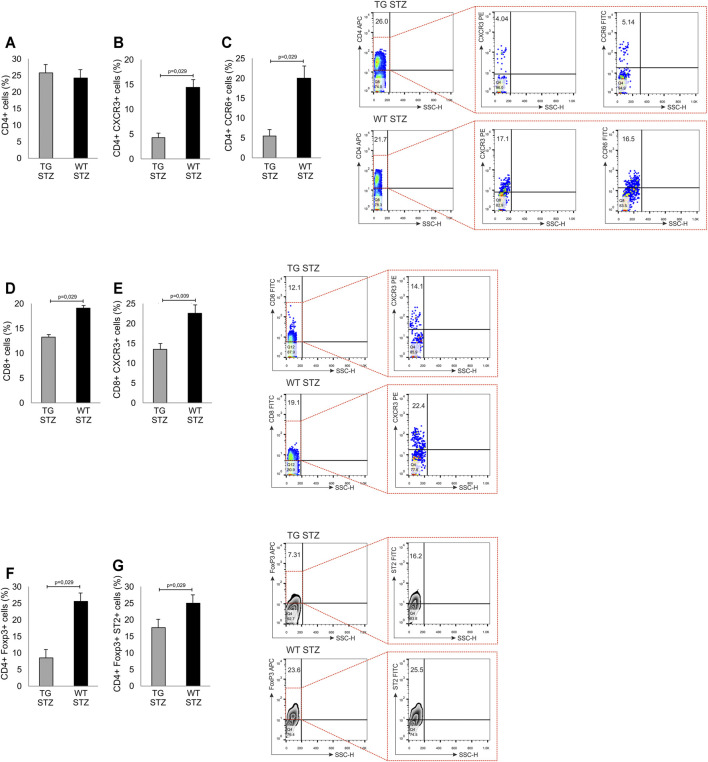
Transgenically enhanced Gal-3 expression on β-cells decreases the percentage of pro-inflammatory cells in pancreatic islets. Phenotypic characteristics of cells in pancreatic islets. Percentage of pro-inflammatory and regulatory cells. The percentages and representative dot and contour plots of **(A)** CD4^+^ cells, **(B)** CD4+CXCR3+ cells, **(C)** CD4+CCR6+ cells, **(D)** CD8^+^ cells, **(E)** CD8+CXCR3+ cells **(F)** CD4+FoxP3+ cells, and **(G)** CD4+FoxP3+ST2+. Data from two individual experiments with at least 5–7 mice per group are shown as mean ± SD and compared by the paired *t*-test or Mann–Whitney test.

In order to investigate the effects of Gal-3 overexpression in β cells on cellular make up of inflammatory infiltrates, we performed a phenotypic analysis of the mononuclear cells in the islets. There was a similar number of mononuclear cells and the percentage of total CD4^+^ T lymphocytes (Figure 4 panel A), with a significantly lower percentage of pro-inflammatory CD4^+^CXCR3^+^ Th1 cells (*p* = 0.029) in islets of TG mice than in the WT (Figure 4 panel B). Also, the percentage of CD4^+^CCR6^+^ Th17 cells was significantly lower in TG mice (Figure 4 panel C; *p* = 0.029). The percentage of CD8^+^ T cells and percentage of effector CD8^+^CXCR3^+^ T cells were significantly lower in TG mice than in WT mice (Figure 4 panel D, E; *p* = 0.029 and *p* = 0.009, respectively). Further analysis showed that the percentage of regulatory FoxP3^+^ T cells and the percentage of ST2^+^ regulatory FoxP3^+^ cells was also lower in TG mice than in the WT mice (Figure 4 panel F, G; *p* = 0.029).

### TG Overexpression of Gal-3 in β Cells Decreases the Number of Pro-Inflammatory Cells in Draining Pancreatic Lymph Nodes

Antigen(s) released after initial β cell destruction with low dose of STZ stimulate inflammatory cells in the draining pancreatic lymph nodes (Hall et al., 2008; Li et al., 2009). We analyzed whether Gal-3 expression on β cells affects the percentage of inflammatory cells in the lymph node. There was no difference in the number and percentage of CD3^+^ cells. However, there was a significantly lower percentage of CD8^+^ T lymphocytes (*p* = 0.04) with a lower percentage and number of effectors CD8^+^ IFNγ^+^ cells (*p* = 0.031, *p* = 0.007, respectively) in TG mice than in the WT mice (Supplementary Material S2 panels B and C). There were also a significantly lower percentage and number of CD4^+^ helper T lymphocytes (*p* = 0.04 and *p* = 0.046, respectively) with a significantly lower number of CD4^+^ IFNγ^+^ T cells (*p* = 0.003) (Supplementary Material S2 panels D and E). A significantly lower number of CD11b^+^ monocytes (*p* = 0.006) and number of pro-inflammatory CD11b^+^ TNF-α^+^ monocytes (*p* = 0.031) as well as the number of F4/80^+^ macrophages (*p* = 0.008), and pro-inflammatory F4/80^+^ IL-6^+^ macrophages (*p* = 0.017) were found in the pancreatic lymph nodes of TG mice (Supplementary Material S2 panels F–I). Thus, the downregulation of inflammatory cells in the pancreatic lymph nodes correlated with the protective effect of overexpressed Gal-3 in pancreatic β cells.

### Exogenous IL-33 Enhances the Number of Tregs in Pancreatic Lymph Nodes of TG Mice

**FIGURE 5:**
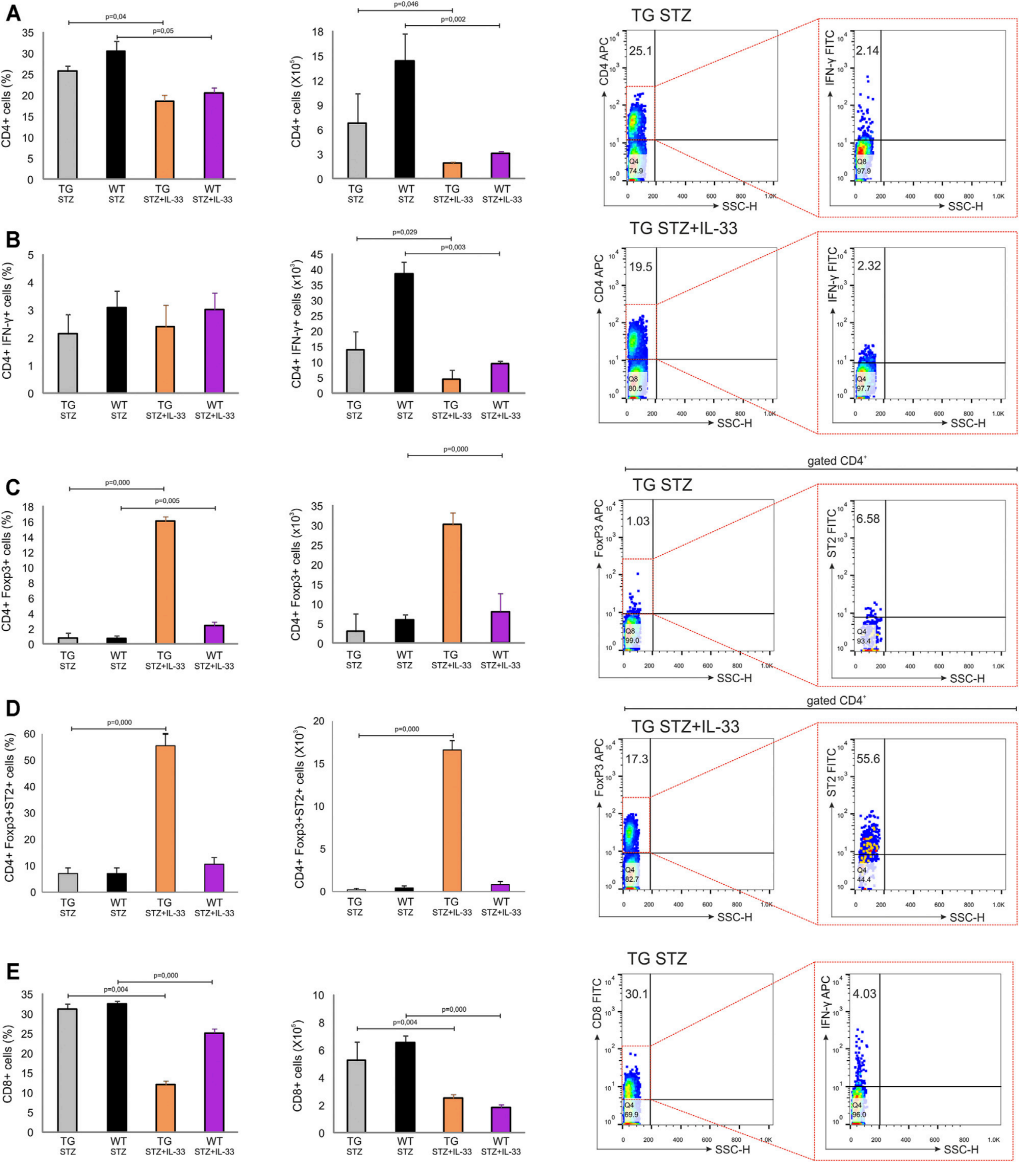
Application of IL-33 (12–18 days) significantly increased the number of Foxp3+ cells in draining pancreatic lymph nodes of TG mice. Differences in number of regulatory and pro-inflammatory cells were observed after administration of multiple low doses of streptozotocin. The percentage, total number, and representative dot plots of **(A)** CD4^+^ cells, **(B)** CD4+IFN-γ+ cells, **(C)** CD4+Foxp3+ cells, **(D)** CD4+Foxp3+ST2+ cells, and **(E)** CD8^+^ cells. Data from two individual experiments with at least 5–7 mice per group are shown as mean ± SD and compared by the paired *t*-test or Mann–Whitney test.

We have shown previously that IL-33 exhibit complete protection of MLD–STZ diabetes if given from the beginning of the disease induction (Pavlovic et al., 2018). However, IL-33 treatment was much less effective if given after the diabetes onset or if applied in NOD mice (Pavlovic et al., 2018). Since Gal-3 overexpression did not affect regulatory T cells in MLD–STZ diabetes in TG mice, we investigated whether the stability of the islet cells and the possible lower level of diabetogenic effect were affected by IL-33. The number of total CD4^+^(*p* = 0.046) and effector CD4^+^INF-γ^+^ (*p* = 0.029) cells was higher in mice not treated with IL-33 (Figure 5 panels A, B). As shown in Figure 5, IL-33 increases the number of FoxP3^+^ T-regulatory cells (*p* = 0.001) even if given late after the disease onset (Figure 5, panel C). Also, the number of ST2^+^ FoxP3^+^ regulatory cells was dramatically higher in TG mice (*p* = 0.000) (Figure 5, panel D).

### Exogenous IL-33 Enhances Protective Effect of Gal-3 Overexpression in the Islets

**FIGURE 6:**
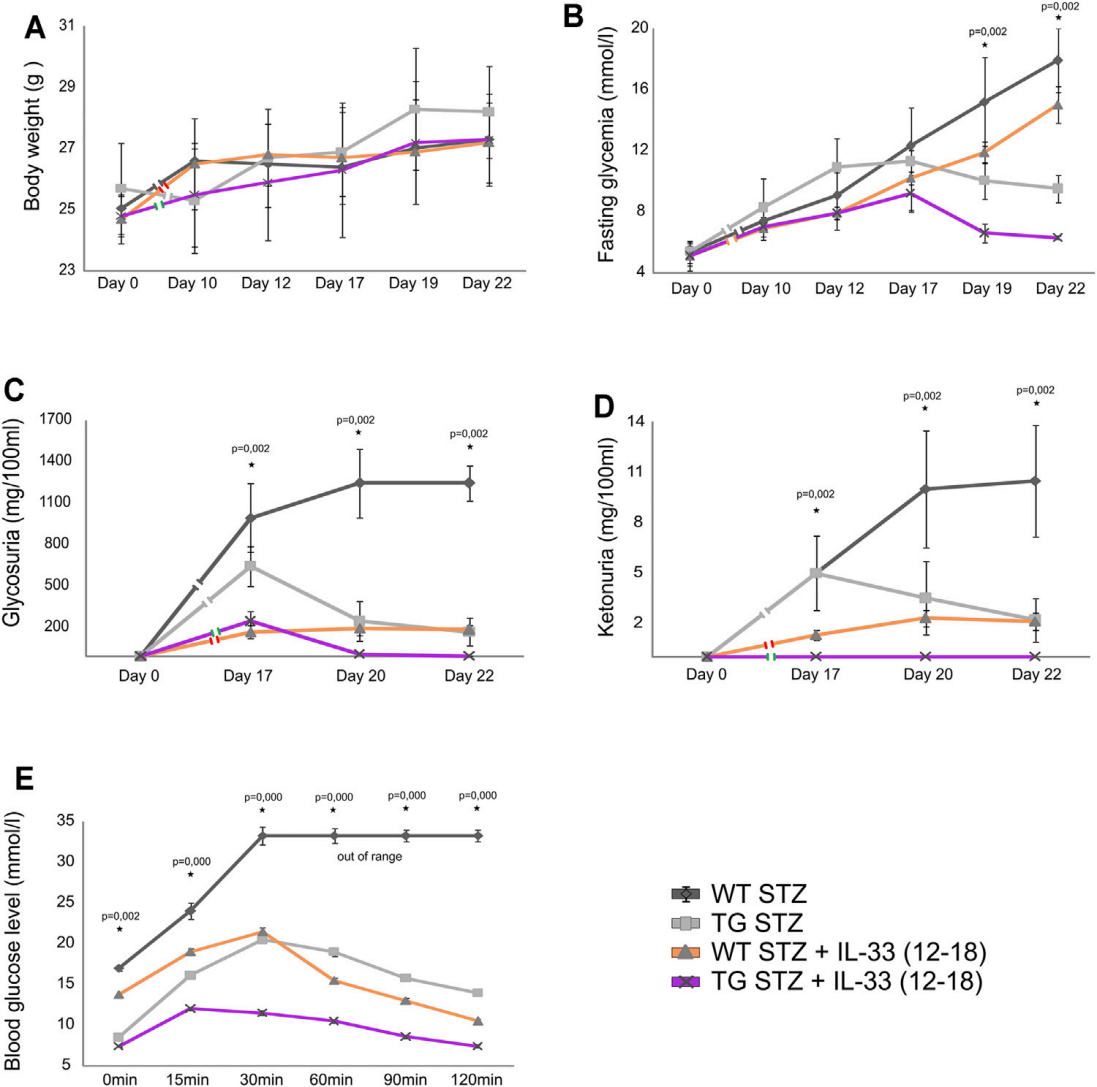
Application of exogenous IL-33 on 12–18 days attenuates the development of hyperglycemia, glycosuria, ketonuria, and hyperglycemia during ipGTT. **(A)** There were no differences in body weight in between TG and WT mice. **(B)** Fasting glycemia was significantly lower in TG mice than in the WT mice. **(C)** Glycosuria was significantly lower in TG mice after the application of IL-33 than in the WT group. **(D)** Ketonuria was significantly lower in TG mice than in the WT mice. **(E)** Glycemia in ipGTT was significantly lower in TG mice than in the WT mice. Data from two individual experiments with at least 5–7 mice per group are shown as mean ± SD and compared by the paired t-test or Mann–Whitney test.

Delayed application of IL-33 after MLD–STZ diabetes induction was not very effective (Pavlovic et al., 2018). Therefore, we tested the combined effect of the delayed, therapeutic administration of IL-33 and Gal-3 overexpression in β cells. As shown in Figure 6, this delayed administration of IL-33 was dramatically more effective in TG Gal-3 mice when compared with the effect in WT mice. Thus, inherent protection from apoptosis, combined with the expansion of regulatory FoxP3^+^ T cells favorably influenced glycoregulation by significantly suppressing fasting glycemia (Figure 6B), glycosuria (Figure 6C), ketonuria (Figure 6D), and glucose tolerance (Figure 6E).

## Discussion

In this article, we have shown that the enhanced Gal-3 expression in β cells shield those cells from immune-mediated damage. These effects are accompanied by the changes in inflammatory cellular composition in the pancreatic drainage lymph nodes as well as in pancreatic islets. The enrichment of regulatory T cells by IL-33, during the development of the disease, demonstrates a synergistic antidiabetic effect with an enhanced Gal-3 expression in β cells.

To study the effect of enhanced Gal-3 expression in β cells *in vivo* on their function in T1DM, we used the experimental model of MLD–STZ diabetes. In the cells, Gal-3 binding to the ligand with its N-terminal domain is at variance with the extracellular Gal-3 use of CRD domain in the induction of inflammation (Nieminen et al., 2005; Robinson et al., 2019).

Normally, in cells, Gal-3 is predominantly found in the cytoplasm (Haudek et al., 2010). It is compounded by the similarity of a part of Gal-3 molecule to the Bcl molecule, which allowed Gal-3 to have an anti-apoptotic effect. In this way Gal-3 can prevent the fatal damage of mitochondria, which further increases the survival chances of cells. This effect directly decreased the intrinsic pathway of cell apoptosis and prolongs their life, allowing better function (Haudek et al., 2010; Harazono et al., 2014). Gal-3 is also considered a potential damage-associated molecular pattern (DAMP) (Sato et al., 2009).

In T cell–mediated autoimmune diseases, the role of Gal-3 appears controversial. Gal-3 knock-out mice display clearly diminished macrophages and T-cell infiltration, and therefore lesser disease severity (Jiang et al., 2009). It has also been shown that Gal-3 deficiency supports the survival and function of beta cells, comprised by the combined effects of TNF-α, IFN-γ, and IL-1β *in vitro* (Saksida et al., 2013). However, it is not clear whether the lack of membranous or intracellular Gal-3 is responsible for this *in vitro* effect. However, the investigation of the role of Gal-3 in the CNS has shown that Gal-3 expression is upregulated in microglia and supports the idea that Gal-3 is a modulator of microglia response to favor the onset of remyelination and oligodendrocyte differentiation (Hoyous et al., 2014). Further, Gal-3 knock-out mice submitted to cuprizone-induced demyelination exhibit higher microglial proliferation and apoptosis as evidenced by the increased activation of caspase-3 which may respond to the well-established Gal-3 anti-apoptotic role (Yang et al., 1996). Thus, it appears that the localization of Gal-3 in the cell and on the cell surface may lead to a different outcomes and cell apoptosis.

We showed that fasting hyperglycemia, ketonuria, and glycosuria are not seen in TG mice and that the TG mice had significantly more insulinemia than WT mice at the end of the experiment (Figure 2). We have previously shown that the disruption of the Gal-3 gene results in decreased susceptibility to MLD–STZ–induced diabetes (Mensah–Brown et al., 2009). This effect is due to the lack of CRD domain leading to the extracellular binding of Gal-3 on antigen-presenting cells and macrophages, and their inflammatory effects. Thus, we assumed that the Gal-3 effect on immune cells did not overcome its anti-apoptotic effect of intracellular Gal-3 in β cells (Yang et al., 1996; Joo et al., 2001; Hsu et al., 2009). Better glycoregulation in TG mice correlates with significantly lower islet infiltration by immune cells (Figure 2 panel A, B, C). INF-γ and TNF-α are major players in β-cell destruction (Rabinovitch and Suarez-Pinzon, 2003). These cytokines are mainly produced by Th1 cells and macrophages (Rabinovitch, 2003). Additionally, IL-17 is involved in organ-specific autoimmunity (Zhu and Qian, 2012), including diabetes (Honkanen et al., 2010; Li et al., 2014). The involvement of CD8^+^ T cells in T1DM is also postulated in humans (Pinkse et al., 2005; Coppieters et al., 2012) and in mice (Yoneda et al., 1997; Wong et al., 2006). The production of all cytokines relevant for the development of T1DM is attenuated in TG mice.

The presence of FoxP3^+^ cells was also increased in TG mice, but the ratio of inflammatory and regulatory cells in the islets were lower in TG mice. In the pancreatic lymph nodes, the number of macrophages and pro-inflammatory accessory cells were also lower in TG mice. Thus, overall binding of Gal-3 in the β cells in TG mice via N-terminal domain, overcome pro-inflammatory effects on antigen presenting and effector immune cells dependent on CRD domain. Analysis of phenotypic characteristics of infiltrating islet cells revealed significantly fewer prodiabetogenic cells in the islets of TG mice. This is especially pronounced in the number and percentage of Th1 and Th17 cells as well as total and activated cytotoxic T cells (Figure 3 panels B, C, D, and E).

**FIGURE 7:**
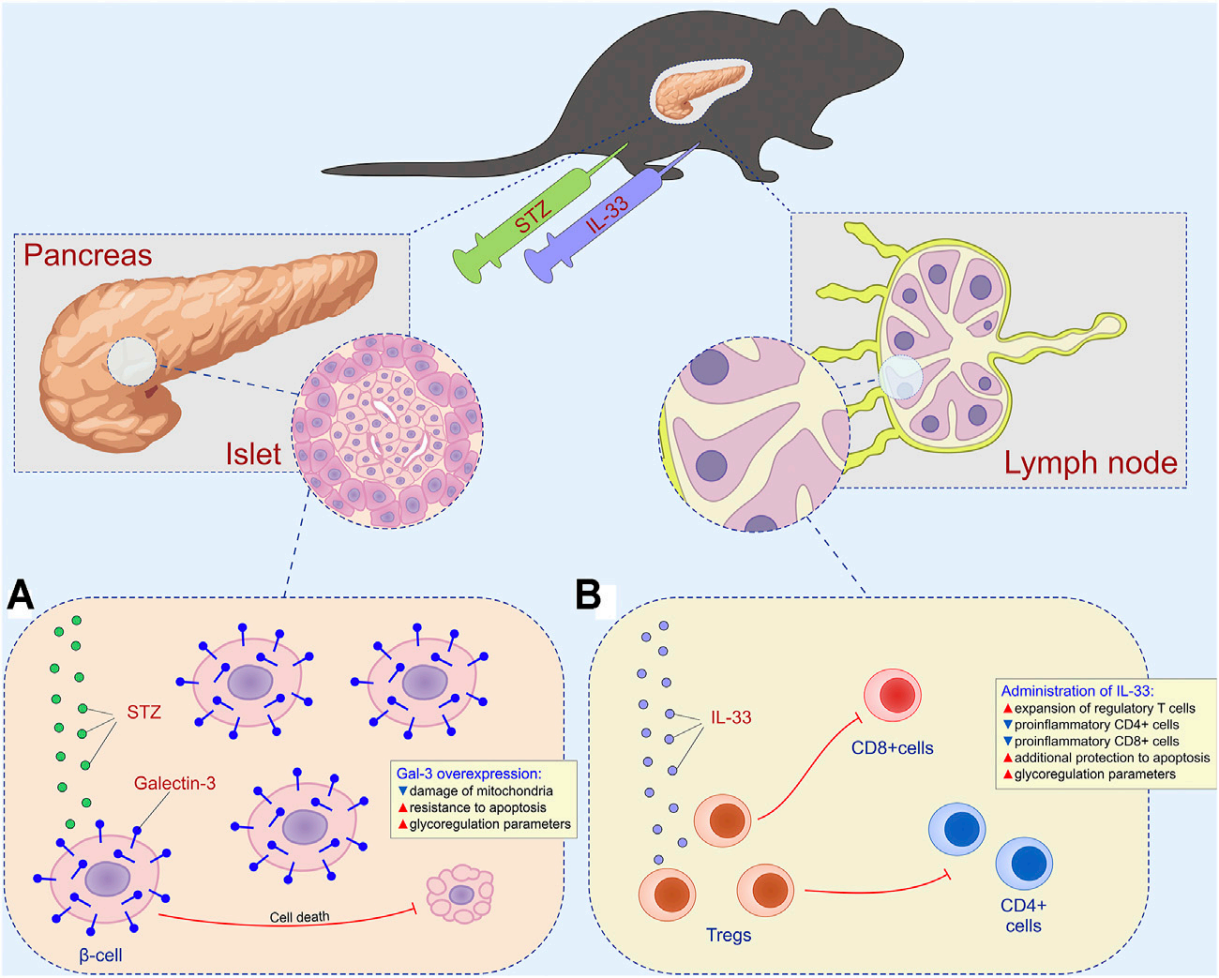
Combined effect of intracellular galectin-3 and IL-33 in STZ-induced diabetes. **(A)** Overexpression of intracellular galectin-3 on beta cells increases the resistance to apoptosis and STZ-induced release of autoantigen. **(B)** Exogenous IL-33 increases the number of regulatory T cells and reduces the number of effector CD4^+^ and CD8^+^ cells.

Our study also showed that the administration of IL-33 after the onset of hyperglycemia has a therapeutic effect in TG mice which was not seen in WT mice. TG mice treated with IL-33 (12–18 days of experiment) were more resistant to MLD–STZ–induced diabetes as evaluated by all metabolic parameters (Figure 6 panel A, B, C, D), particularly in the glucose tolerance test (Figure 6 panel E), compared to the TG mice without IL-33 treatment. We also showed the increase in percentage and the number of regulatory T cells in draining pancreatic lymph nodes (Figure 5 panel C) and lower total pro-inflammatory cells (Figure 5 panel B, Figure 7). Several authors including us (Pavlovic et al., 2018; Lu et al., 2019; Desbois et al., 2020; Lu et al., 2020) have shown that the administration of IL-33 had a significant effect on the increased regulatory FoxP3^+^ cells, which suppress autoimmune diseases including the MLD–STZ diabetes (Pavlovic et al., 2018).

Figure 7 depicts the combined effects of overexpressed Gal-3 and the administration of exogenous IL-33 on the development of hyperglycemia and diabetic state. Overexpression of intracellular Gal-3 downregulates apoptosis (Figure 7A), while IL-33, by stimulating regulatory T cells, downplays islet inflammation and beta-cell loss induced by CD4^+^ and CD8^+^ cells and their pro-inflammatory mediators (Figure 7B). Thus, in this article, we provided evidence that not only the enhanced expression of Gal-3 in β cells reduced T cell–mediated autoimmune inflammatory disease but also that exogenous IL-33 application had a powerful therapeutic effect in TG mice. Furthermore, the Gal-3 overexpression in β cells significantly reduced mononuclear infiltration in the islets, and IL-33 enhanced this effect.

## Data Availability

The raw data supporting the conclusion of this article will be made available by the authors, without undue reservation.
