# Supplementary material for: Transgenic Overexpression of Galectin-3 in Pancreatic β Cells Attenuates Hyperglycemia in Mice: Synergistic Antidiabetic Effect With Exogenous IL-33
Source: Front Pharmacol. 2021 Nov 5;12:714683. doi: 10.3389/fphar.2021.714683 (PMC8602837; doi:10.3389/fphar.2021.714683)
Supplement: Supplementary file 1 [file Table1.DOCX]

Supplement 1.

| Primers (Kapa Biosystems, USA) | |
| --- | --- |
| Forward | ATGCTCAGCCAAGGACAAAG (On RIP Promoter) |
| Reverse | AGTTGGGCCAGGATAAGCTC (On Lgals3 cDNA) |

Supplement 1: PCR confirmation of genotype. PCR reaction was performed using specific set of primers (Kapa Biosystems, USA) and the presence of a 591bp product was visualized on agarose gel.
