# Supplementary figures and images for: Transgenic Overexpression of Galectin-3 in Pancreatic β Cells Attenuates Hyperglycemia in Mice: Synergistic Antidiabetic Effect With Exogenous IL-33
Source: Front Pharmacol. 2021 Nov 5;12:714683. doi: 10.3389/fphar.2021.714683 (PMC8602837; doi:10.3389/fphar.2021.714683)

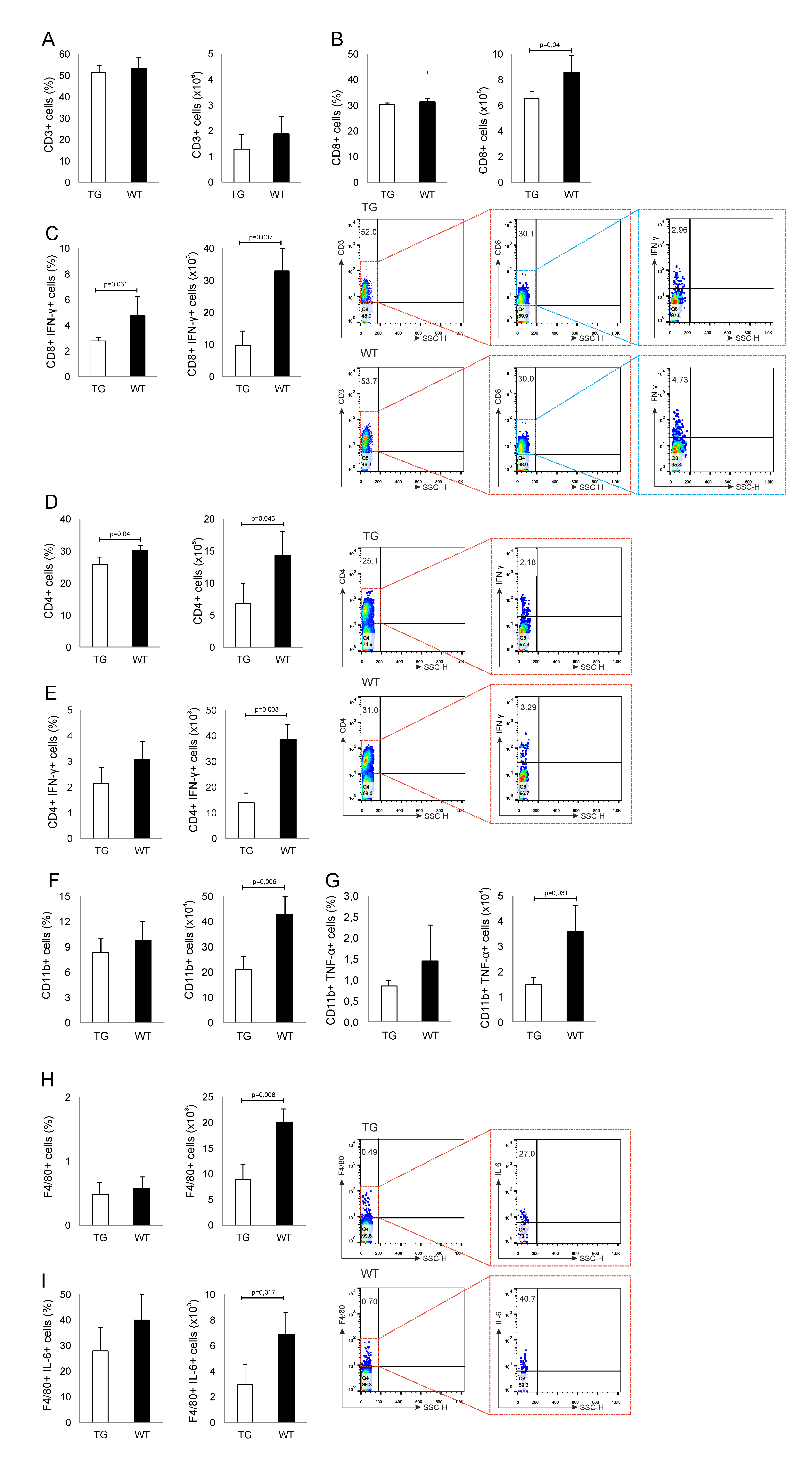

Supplement: Supplementary file 2 [file Image1.TIF]
